# Contrasting geographic patterns of parasite and hantavirus diversity in the rodent *Oligoryzomys longicaudatus* (Rodentia, Cricetidae)

**DOI:** 10.1371/journal.pntd.0014424

**Published:** 2026-06-26

**Authors:** Reilly N. Brennan, R. Eduardo Palma, Sally L. Paulson, Wendy C. Hernández-Mazariegos, Luis E. Escobar

**Affiliations:** 1 Department of Entomology, Virginia Tech, Blacksburg, Virginia, United States of America; 2 Facultad de Ciencias Biológicas, Pontificia Universidad Católica de Chile, Santiago, Chile; 3 Universidad de O’Higgins, Rancagua, Chile; 4 One Health Institute, Faculty of Life Sciences, Universidad Andres Bello, Santiago, Chile; 5 Department of Fish and Wildlife Conservation, Virginia Tech, Blacksburg, Virginia, United States of America; 6 Center for Emerging, Zoonotic and Arthropod-borne Pathogens, Virginia Tech, Blacksburg, Virginia, United States of America; 7 Global Change Center, Virginia Tech, Blacksburg, Virginia, United States of America; University of Texas Medical Branch, UNITED STATES OF AMERICA

## Abstract

Host–parasite interactions arise from a complex interplay of evolutionary history, ecological context, and community structure, yet these dimensions are rarely examined together. Here, we introduce a unified framework that links the macroevolutionary processes shaping host–parasite associations with the microevolutionary dynamics driving intraspecific viral and host diversity. This approach reveals how evolutionary and ecological forces jointly structure parasite and viral diversity across a host’s range. We used the hantavirus host *Oligoryzomys longicaudatus* in South America as a model system to explore this analytical framework. The objective of this study was to uncover potential factors contributing to parasite and viral diversity in this system in a framework that can be applied to other disease systems. Our data suggest that parasite richness peaks in environmentally optimal, central regions of the host’s range, while hantavirus diversity peaks toward environmental and geographic margins. This dynamic connection among host ecology, parasite community turnover, and viral evolution illustrates how geographic and environmental variation influence host-parasite evolution. By bridging micro- and macroevolutionary signals, our analytical framework provides a biologically sound approach for describing host, parasite, and viral diversification in a changing world, and a foundation to explain how diseases emerge across changing landscapes.

## Author summary

In nature, parasites, the animals they infect, and the environments they share shape one another in complex ways. These interactions can create different patterns of parasite diversity and influence where diseases are more likely to occur. Factors such as an individual animal’s genetics, the community of species it interacts with, and broad environmental conditions play important roles in transmission. In this study, we wanted to understand how these pieces fit together in a wild rodent of public health significance. We developed an approach that links these different scales and applied it to the long-tailed mouse in South America to examine the drivers of both hantavirus and parasite diversity. We found that parasite diversity was highest in the central part of the host range, where environmental conditions are more favorable, while viral diversity peaked at the edges of the host range. These contrasting patterns show that parasites and viruses can respond differently to the same hosts geographic history and environmental pressures. By combining genetic, ecological, and environmental information, our work helps clarify how disease systems function and how they may shift as landscapes and climate change.

## Introduction

Parasites often exhibit a preference for specific host species, a phenomenon known as host specificity, which shapes host-parasite dynamics and disease emergence [1,2]. Both endoparasites (e.g., viruses and bacteria) and ectoparasites (e.g., ticks, fleas, and mites) commonly exhibit host specificity, reflecting extensive variability of host use across parasite taxa [1,3,4]. Notable examples of host specificity include both vector-borne and directly transmitted parasites across diverse taxonomic groups like *Plasmodium* (malaria parasites), *Bartonella* (flea-borne bacteria), *Trypanosoma* (protozoa), *Ixodes* ticks (vectors for the Lyme disease causing bacteria), and host-specific viruses like hantavirus, rabies virus, and pox viruses [3,5–7].

Most zoonotic parasites, which are human disease-causing parasites of animal origin, have a broad host range but often originate from a ubiquitous, single wildlife host species [8]. Parasite specificity to hosts can be both a necessity and a constraint for parasite success [1]. Thus, host-parasite relationships are often shaped by co-evolutionary processes, in which hosts and parasites exert reciprocal selective pressures [9]. Co-evolutionary theory of host-parasite relationships identifies host characteristics, parasite characteristics, and the environment as regulating the success of host-parasite interactions [9]. Characteristics of hosts, parasites, and the environment, however, are not uniform along the distribution of a host, resulting in variability of host-parasite interactions throughout a host’s distribution. Although these co-evolutionary dynamics can be difficult to validate empirically, they form a conceptual framework crucial to understanding parasite ecology, evolution, and the potential for cross-species transmission, including zoonotic spillover to humans [9,10].

Parasite adaptation and success are influenced by a combination of host, environmental, and geographic characteristics spanning macro- to micro-ecological and evolutionary scales [11–14]. Host characteristics and genetic diversity reflected in immune system complexity, behavior, social structure, and preferred habitat can help explain parasite specificity and likelihood of cross-species transmission [9,11,12]. Environmental conditions, such as temperature, humidity, and seasonal variability, influence parasite development, survival, and transmission [15]. Geographic distribution defines the spatial overlap between hosts and parasites and their distributional boundaries, shaping the likelihood of interaction and the potential for parasite transmission [16]. The interplay among host, environment, and geographic factors determines the likelihood of parasite establishment within one and multiple hosts and the broader patterns of parasite diversity [14].

Parasite species assemblage is considered on a hierarchical scale [16]. Parasites found on a single individual host are referred to as the ‘infracommunity’, within a host population as ‘parasite community’, and within the entire host species as ‘parasite fauna’ [16]. Parasite species richness has been studied across host systems, temporal and geographic scales, and host hierarchies in multi-host systems [14,17,18]. Notable findings include that parasite species richness is positively associated with host density [19], range size, and body mass in carnivores [20], host fitness in birds [21], and lower latitudes for rodents [22]. Host body size, geographical range size, and density have been the most consistent predictors of parasite species richness among animals, plants, and fungi [23]. It is still unclear, however, how parasite fauna in a single host species varies throughout the host range. For host species that harbor parasites of medical or veterinary concern, variation in parasite fauna can reveal the drivers of host susceptibility and inform surveillance within a host species. Studying the drivers of parasite fauna in a single host could offer insights into host history [24] and could provide foundational theory for patterns observed across host species assemblages. A model host system to address these gaps would need a broad geographic range to cover diverse environmental gradients and a comprehensive understanding of its parasite fauna.

Andes virus (ANDV; *Orthohantavirus andesense*) is a hantavirus of broad distribution in South America that poses a significant public health risk due to its associated disease severity and unique viral characteristics [25]. Each year, there are approximately 120 human cases of hantavirus cardiopulmonary syndrome (HCPS) caused by Andes virus [25]. Of all hantaviruses, Andes virus is among the most severe with 21–40% mortality [25,26] and has evidence of human-to-human airborne transmission [27]. Primarily responsible for transmission to humans is the rodent host, *Oligoryzomys longicaudatus,* also known as “colilargo” or the long-tailed pygmy rice rat [28]. Aside from the Andes virus, *O. longicaudatus* also serves as the host for a plethora of endo- and ecto-parasites [29]. In Chile, *O. longicaudatus* shares ectoparasite species with many other rodent hosts, playing a key role in maintaining parasites diversity within rodent communities [30]. *Oligoryzomys longicaudatus* is distributed throughout most of Chile and into parts of central Argentina [31] but ancestrally originated in the southern temperate forests of Chile and eastern Argentina [32]. The population density of *O. longicaudatus* is influenced by diverse ecological factors and has been temporally linked to *Chusquea* spp. bamboo flowering [33] and El Niño–Southern Oscillation [33,34]. Previous studies have found that rodent ANDV seropositivity is higher in the center of *O. longicaudatus*’ distributional range, while human cases are mostly in its southern range, indicating a mismatch between where rodent seropositivity occurs and where human cases are reported [35]. Considering the public health concerns associated with *O. longicaudatus*, as well as its broad geographic and environmental distribution, it is a prime study host for assessing the distribution of both interspecific parasite fauna and intraspecific variation in Andes virus.

In this study, we address fundamental questions about the ecology and evolution of parasite diversity along optimal and suboptimal environmental gradients of the host and throughout its geographic distribution. We address why and how parasite diversity varies along a single host’s distribution and compare interspecific parasite vs. intraspecific viral diversity from a single host. We develop a multi-scale framework that links macro- to micro- ecological and evolutionary factors which contribute to host-parasite interactions with trends in parasite and viral diversity through their connection in geographic and environmental space. Through this, we uncover geographic and environmental factors associated with host-parasite interactions and identify potential mechanisms of parasite diversification for further exploration. We include the evolutionary history of both the host and its parasites to improve our theoretical [36,37] and analytical [38] frameworks and further refine eco-evolutionary approaches in disease ecology [37]. We hypothesize that parasite species richness and phylogenetic richness of ANDV carry a geographic and environmental signal that can be detected and reconstructed along the host’s distributional range.

## Materials and methods

**Fig 1:**
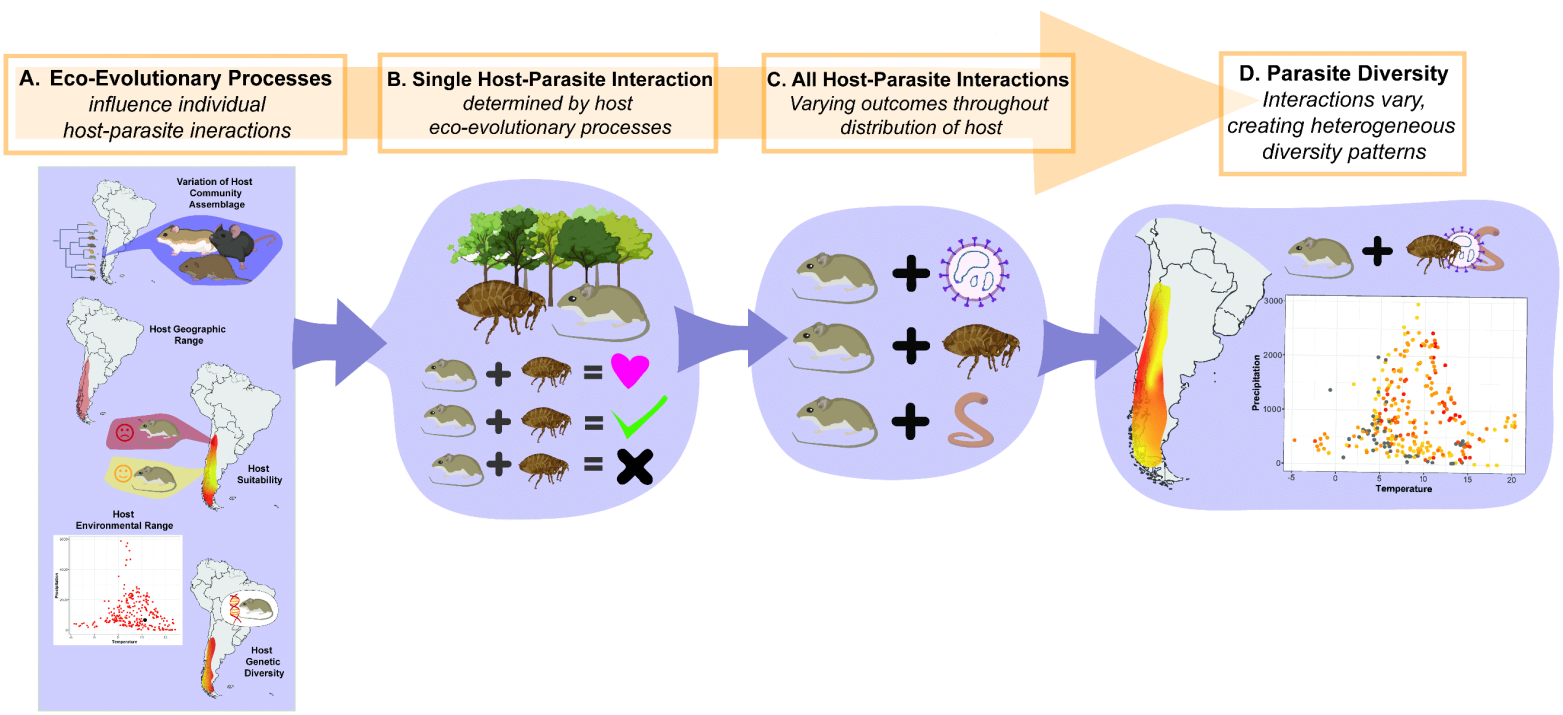
Framework for Accumulation of Parasite Diversity. **(A)** Host-parasite interactions in the present are the result of a macro- and micro- ecological and evolutionary pressures. These pressures include host assemblage (i.e., host species richness), host geographic range, host environmental suitability, host environmental range, and host intraspecific genetic diversity. **(B)** The latter pressures influence whether a single parasite will 1. encounter and 2. have success infecting a host. Parasite species may be able to infect a given host to varying degrees of success. **(C)** The outcome of all host-parasite interactions varies greatly throughout the distribution of a host depending on which lead to **(D)** spatial heterogeneity in parasite diversity (both intra- and inter- specific) for a given host both in geographic and environmental space. Our framework links the micro and macro processes from panel A to the observed patterns in parasite species richness in panel D by studying these factors collectively in geographic and environmental space. This fig was created in Adobe Illustrator software with graphics from BioRender. Cifuentes-Rincon, **A.** (2026) https://BioRender.com/2w7fcq4. The base map for Argentina and Chile was downloaded from DIVA-GIS (https://diva-gis.org/data.html) with data provided by GADM under license CCBY (https://gadm.org/license.html).

We sought to determine the drivers of interspecific parasite species richness and of intraspecific viral genetic diversity. First, we collected data associated with the rodent *O. longicaudatus* to estimate the parasite species richness and viral genetic diversity throughout its distribution. In our framework (Fig 1) we propose that a combination of macro- and micro- ecological and evolutionary factors may influence parasite diversity (both interspecific parasite species richness and intraspecific viral diversity). We estimate host community assemblage, host geographic range, host environmental suitability, host environmental range, and host genetic diversity and link them through geographic and environmental space to measures of interspecific parasite species richness and intraspecific ANDV phylogenetic richness (Fig 1).

### Data acquisition and review

We defined parasites as organisms feeding or relying on a host for survival, including ectoparasites (ticks, mites, fleas) and endoparasites (bacteria, viruses, protozoa, worms, nematodes). We recovered records of parasites and genetic sequences associated with *O. longicaudatus* or ANDV (search date: January 7, 2024). Data repositories screened included Web of Science Core Collection, Zoological Record from Web of Science, Wildlife and Ecology Studies Worldwide from EBSCOhost, PubMed from NLM, and Biological Abstracts from Clarivate Analytics with the search terms, “Oligoryzomys longicaudatus” OR “Colilargo*” OR “long-tailed pygmy rice rat” OR “colilargo*” in Title/Abstract. Articles were excluded if they did not directly trap *O. longicaudatus*, did not test for pathogens, parasites, or genetic material, were literature reviews, geographic location was missing, or were studies in humans. Using Covidence software [39] we reviewed each result and extracted species names and prevalences of parasites, geographic location, and Genbank accession numbers.

### Phylogenetic tree reconstruction

From the sequences found through the literature review we generated evolutionary histories for *O. longicaudatus* and ANDV using the loci with the most sequences. We obtained sequences from NCBI Genbank (www.ncbi.nlm.nih.gov/genbank) by downloading the .fasta files from the web interface and imported them into RStudio Version 2024.04 [40], using the *Biostrings* package (Pagès et al., 2024). We aligned all sequences using the MUSCLE algorithm with standard parameters provided by the function ‘msaMUSCLE’ in the R package *msa* [41,42]. MUSCLE was selected for its high accuracy and computational efficiency in multiple sequence alignment [42]. To minimize bias due to unequal sequence length and poorly aligned regions, we trimmed the alignments using the ‘msaTrim’ function from the *microseq* package [43] removing regions with >20% gaps at the ends and >90% gaps within the alignment. Phylogenetic trees were inferred using a maximum-likelihood framework from the *phangorn* package [44]. We used the ‘modelTest’ function to compare substitution models and selected the best-fitting model based on the Bayesian Information Criterion, which balances model fit with parameterization. This model was used to construct an initial tree using the ‘pml_bb’ function, inferring a tree with maximum likelihood. To evaluate the robustness of the phylogenetic relationships, we conducted a non-parametric bootstrap analysis with 1000 replicates using ‘bootstrap.pml’. The number of replicates was chosen to balance computational efficiency with statistical reliability [45]. The consensus tree was generated from the bootstrap replicates, retaining branches supported by at least 50% of the samples, to reflect moderate-to-strong support while avoiding overfitting low-confidence nodes.

### Nucleotide and haplotype diversity

We calculated nucleotide and haplotype diversity for *O. longicaudatus* using the same sequences from phylogenetic richness analysis to identify population-level genetic variation, recent evolutionary processes, and demographic history [46]. We calculated nucleotide and haplotype diversity using the R package *pegas* [47]. Nucleotide diversity (π) was calculated as the sum of the number of differences between sequence pairs divided by the total number of comparisons [48]. Haplotype diversity (*h*) was calculated as the probability that two randomly chosen haplotypes are different [49]. We calculated nucleotide and haplotype diversity using spatial balanced clustering with the R package *anticlust* [50].

### Mapping phylogenetic richness and genetic diversity

To map phylogenetic richness for *O. longicaudatus* and ANDV, we used a 0.5-degree (~27 km) radius from the sampling site of each genetic sequence as a proxy of an individual-rodent home range [51,52]. We calculated phylogenetic diversity with the R package *phyloraster* [53] which uses the sum of branch lengths for all records in each region, as defined by the raster resolution and dimensions. We defined phylogenetic richness [54] as the average phylogenetic diversity [55] in each site (i.e., raster cell). We divided the phylogenetic diversity calculated for each pixel by the number of individuals in that pixel to obtain an average phylogenetic richness measurement. We mapped nucleotide and haplotype diversity in geographic space for each population cluster of hosts. Population clusters were mapped using the centroid of the cluster estimated by averaging the latitude and longitude from the 14 individuals in each of the 17 clusters. This generated a single point per cluster for which nucleotide and haplotype diversity were assessed.

### Mapping parasite species richness

To map parasite species richness in geographic space, we obtained published records of parasites associated with *O. longicaudatus* from our literature review. We converted all locality and geographic coordinates to decimal degrees from the parasite records using the data provided in each publication or associated supplementary materials. We calculated the parasite species richness associated with *O. longicaudatus* using the ‘rast.sr’ function in the R package *phyloraster* which calculates species richness using a raster file as the number of species present in each raster cell [53].

### Host species richness

We examined the richness of rodent species found in the distribution of *O. longicaudatus* as an assessment of how the larger host community structure influences richness of parasites in *O. longicaudatus*. Most of the *O. longicaudatus* distribution is located Chile, which is a well delimited biogeographic zone in the north (i.e., Atacama Desert), south (i.e., ice field), east (Andes Mountains), and west (i.e., Pacific Ocean) [56], and was thus used as study area. We collected rodent species richness and distributional data from published records in Chile [56] and converted the rodent species richness to raster using the ‘rast.sr’ function in the R package *phyloraster* to calculate species richness using a raster file as the number of species present in each raster cell [53].

### Geographic and environmental space

We examined how host genetic, parasite species richness, and ANDV phylogenetic richness were distributed along geographic and environmental gradients occupied by *O. longicaudatus* by using metrics of centrality for both geographic and environmental gradients. To define geographic space, we downloaded occurrences of *O. longicaudatus* from the Global Biodiversity Information Facility (GBIF) [57] in R using *rgibf* [58]. We constructed a distribution map from the occurrences using the package *gbif.range,* which creates an estimated distribution based on occurrence points and ecoregion polygons [59]. We used this estimated distribution to calculate the centroid in the geographic range using the R package *sf* and function ‘st_centroid’ [60]. We then calculated the Haversine distance from the geographic centroid to each parasite species richness, ANDV phylogenetic richness, and host genetic diversity record using the ‘distm’ function from the package *geosphere* [61].

To define the environmental space, we gathered environmental variables associated with our records of parasite species richness, ANDV phylogenetic richness, and host genetic diversity from WorldClim 2.0 [62] into R using the package *geodata* [63]. We considered mean annual temperature and annual precipitation to represent the environmental gradient occupied by *O. longicaudatus*. To assess how parasite species richness, ANDV phylogenetic richness, and host genetic diversity were distributed in environmental space, we calculated the distance from each record to two different environmental centroids (mean annual temperature and annual precipitation). The two centroids we assessed included a proxy of the realized niche centroid and a proxy of the existing fundamental niche [64,65].

The ecological realized niche refers to the environmental conditions that a species can exist in in the presence of competitors or other restrictive factors [66]. To build the ecological realized niche, we extracted mean annual temperature and annual precipitation WorldClim values for each *O. longicaudatus* occurrence derived from GBIF and our literature review. We calculated the centroid of the realized niche in R using the mean temperature and precipitation values from the occurrences. We measured the Euclidean distance between the realized niche centroid with each *O. longicaudatus* sequence record from our review in R using the function ‘dist’ in the package *stats* [67].

The existing fundamental niche refers to the currently existing subset of environmental space where the species can maintain stable populations without the need for migrants [64]. To assess the existing fundamental niche centroid [64] of *O. longicaudatus*, we generated an ecological niche model of the species using Wallace version 2.2.0. Wallace is a user-friendly R-based Shiny application that allows users to develop highly parameterized Maxent ecological niche modeling [68]. In Wallace we uploaded the GBIF occurrence points used to build the geographic distribution and our literature review occurrences with environmental predictor variables from WorldClim. We used 15 bioclimatic variables available through WorldClim that summarize temperature and precipitation as environmental predictor variables at a 2.5 arcmin resolution [62]. We excluded bioclimatic layers bio 8–9 and 18–19 due to their incorporation of temperature and precipitation in the same layer which can introduce artifacts to the data [69,70]. Within Wallace, we thinned our occurrences first to exclude occurrences falling outside the accepted distribution for *O. longicaudatus*. We considered the distribution for *O. longicaudatus* to extend from 27° to 54° S in Chile [32,71], and into regions of Argentina [72]. Then we thinned occurrences using a 9.05 km radius following the maximum home range size for *O. longicaudatus* [52]. We used a minimum convex polygon with a two-degree distance as a background extent (i.e., study area M) [66]. Remaining occurrence data were partitioned into training and validation data with a block spatial partitioning method (k = 4 folds) [73]. In Wallace, we used the Maxent algorithm to build an ecological niche model to estimate environmental suitability. Maxent was selected due to its ability to work with presence-background data and proven reliability as a modelling algorithm [74,75]. We used linear, linear-quadratic, and hinge feature classes, and regularization multipliers between 0.5 and 2 and 0.1 step intervals [68,73]. We selected the best model based on minimizing (i) the omission rate as a proxy of failure in predictions and (ii) Akaike information criterion score, which balances model fit and complexity, as a proxy of a model capacity to resemble the relative calibration data. Suitability was assessed on a scale from 0 to 1 using Maxent logistic output. Finally, the Maxent ecological niche model was binarized and analyzed in environmental space to identify the geometric center of the ecological existing fundamental niche, interpreted as the theoretical optimum of the species [76]. The Maxent model was binarized using a ten-percentile threshold and the resulting model was displayed in environmental space of mean annual temperature and annual precipitation to determine the center of the estimated existing fundamental niche. Distance to the center of the existing fundamental niche was measured for each *O. longicaudatus* record using Euclidean distances. We repeated the analysis of suitability and centroids using all GBIF records with records of *O. longicaudatus* from our literature review. We ran this separate model given that the literature reported outlier occurrences outside the accepted distribution of *O. longicaudatus*. We emphasize that the proxy of the existing fundamental niche used in this analysis represents statistical estimates of climatic suitability based on occurrence data and coarse scale environmental data instead of direct measures of fitness or biological optima [77].

### Modeling predictors of diversity

To assess associations between parasite species richness, ANDV phylogenetic richness, and *O. longicaudatus* genetic diversity metrics with environmental and geographic predictors, we first tested our data for normality using ‘shapiro.test’ in the R package *stats* [77]. We then used Spearman’s rank correlation to assess bivariate relationships using ‘cor.test’ in the R package *stats* [66]. We evaluated correlations with mean annual temperature (°C), annual precipitation (mm), logistic suitability score (0–1), rodent species richness, distance from centroid of geographic range (m), distance to realized niche centroid (Euclidean), and distance to existing fundamental niche centroid (Euclidean).

Next, we also sought to determine the most contributing factors to parasite species richness and ANDV genetic diversity by using multivariate modelling. Prior to multivariate modeling, we assessed spatial autocorrelation in regression model residuals using Moran’s I statistic with k-nearest neighbors’ spatial weights (k = 4), implemented in the R packages *spdep* [78]. We fit ordinary least squares (OLS) regression models for parasite species richness and ANDV phylogenetic richness as functions of the environmental and host-related predictors, then tested the residuals for spatial autocorrelation. Initial OLS models showed significant positive spatial autocorrelation in residuals (parasite species richness: Moran’s I = 0.42, p = 0.001; ANDV phylogenetic richness: Moran’s I = 0.22, p = 0.001), violating the assumption of residual independence.

To address spatial autocorrelation, we implemented generalized additive models (GAMs) using the R package *mgcv* [79]. Environmental and host-related predictors included in the GAMs were host phylogenetic richness, environmental suitability, mean annual temperature, annual precipitation, distance to geographic centroid, distance to realized niche centroid, and rodent species richness. Each model included a two-dimensional thin plate regression spline smooth term for geographic coordinates (longitude, latitude) to capture spatial dependence. The basis dimension (k) of the spatial smooth controls model flexibility: higher k values allow more complex spatial patterns but can risk overfitting. We selected k values that balanced model fit with biological reality and tested multiple specifications: the primary model with k = 50 and a sensitivity analysis with k = 30 [79]. For parasite species richness (count data with overdispersion, variance/mean ratio = 4.2), we used a negative binomial error distribution. For ANDV phylogenetic richness (continuous, right skewed data), we used a Gaussian distribution. Model parameters were estimated using restricted maximum likelihood (REML). Post-hoc Moran’s I testing confirmed that the spatial smooth terms resolved residual autocorrelation in both models (parasite SR: Moran’s I = 0.05, p = 0.15 for k = 50; ANDV: Moran’s I = -0.12, p = 0.96 for k = 30).

The narrow distribution of *O. longicaudatus* along the Chilean and Argentine Andes (spanning >2,500 km but <200 km wide) creates spatial structure where environmental gradients are strongly aligned with geographic distance. This makes complete statistical separation of spatial and environmental effects challenging [80,81]. The spatial smooth terms in our GAMs capture geographically structured variation, which may include unmeasured environmental heterogeneity not captured by bioclimatic variables, dispersal limitation and isolation-by-distance, and historical biogeographic patterns.

### Collinearity and sampling bias

We assessed collinearity among predictors using variance inflation factors (VIF) calculated with the *car* package [82]. Precipitation showed moderate collinearity with distance to niche centroid (VIF = 5.6 for SR, 7.7 for ANDV model; Pearson r = 0.81), reflecting the strong latitudinal precipitation gradient across Chile. No VIF exceeded the conventional threshold of 10 indicating severe collinearity, so all predictors were retained in the models [83].

We gathered parasite species richness data from a literature review, which may introduce sampling bias into our data. To address this bias in the literature, we assessed the sampling effort variability and sampling adequacy for our dataset. Sampling effort varied spatially (coefficient of variation = 92%), which is inherent to literature-derived biodiversity data. To assess sampling adequacy, we conducted species accumulation analysis using the *vegan* package [84]. The species accumulation curve reached an asymptote, and the Chao richness estimator suggested 54.8 species (vs. 35 observed), indicating that sampling captured approximately 64% of the regional parasite fauna. We emphasize that the spatial smooth term in our GAMs implicitly accounts for any spatially structured sampling heterogeneity, as it absorbs all spatial variation regardless of its origin.

## Results

### Records of parasites and pathogens

From our literature review, we obtained 154 unique records of *O. longicaudatus* infection with parasites from 1952 to 2019 (S2 Table). We also obtained 69 unique date-location reports of ANDV in *O. longicaudatus* with 2 M segment sequences and 30 S segment sequences available (S4 and S5 Tables). For *O. longicaudatus*, cytochrome b was the locus with the most sequences available, with 237 sequences available (S3 Table). In total, we obtained 391 unique locality occurrences of *O. longicaudatus* with parasite or genetic data (S4 and S3 Tables). In our literature review, we found records of parasites and genetic data attributed to *O. longicaudatus* in northern Chile and northwestern Argentina, where the species is not formally believed to be distributed (S2 and S4 Tables). To mediate these discrepancies, we conducted two suitability analyses- one which operated under the accepted distribution of *O. longicaudatus*, reported in the main text, and one which included all occurrences from both online databases and the literature from Chile and Argentina, reported as supplemental materials (S1 Fig).

### Geographic and environmental space, and suitability

**Fig 2:**
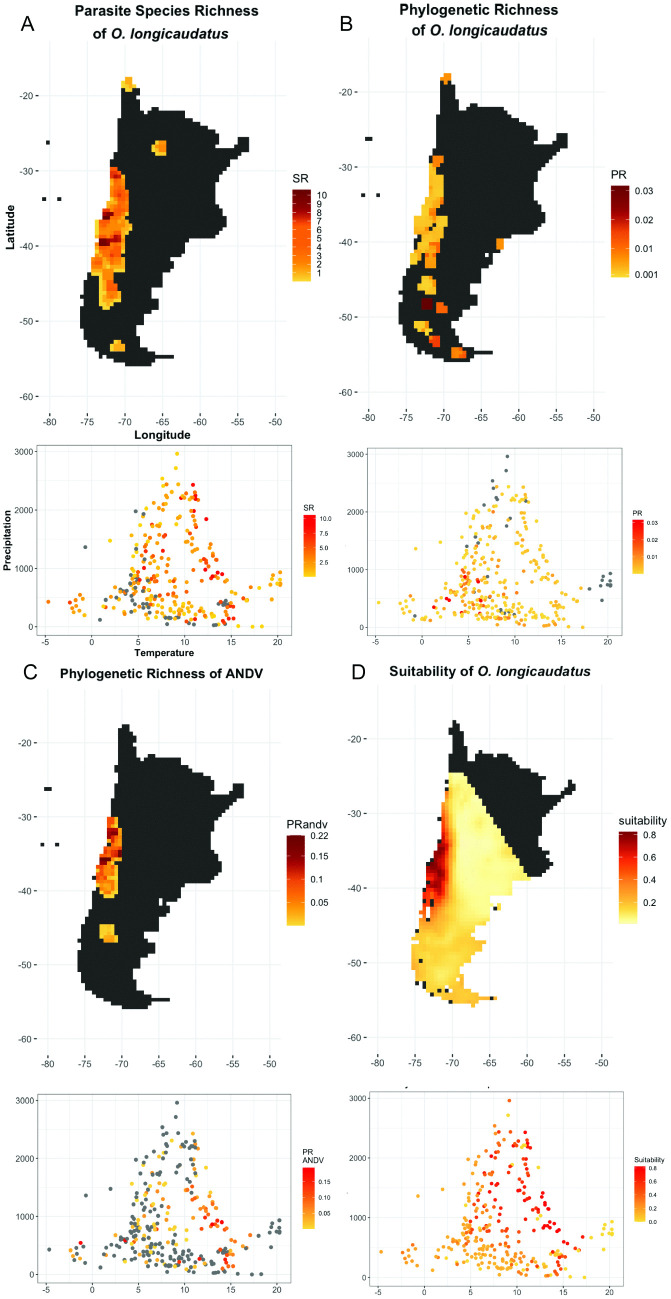
Geographic and environmental space of Parasite Species Richness (parasite species richness), Phylogenetic Richness (phylogenetic richness), Andes Virus (ANDV) phylogenetic richness, and environmental suitability for *Oligoryzomys longicaudatus.* **(A)** parasite species richness is a measure of the number of parasites associated with *O. longicaudatus* in each area and is concentrated in the center of the rodent’s geographic distribution (top), reaching peaks in environmental areas with higher precipitation and warmer temperatures (bottom). **(B)** phylogenetic richness of *O. longicaudatus* is highest at its southern extent, with some pockets of higher richness in the most northern areas of its geographic distribution (top) and is highest in environments with low temperature and precipitation (bottom). **(C)** ANDV phylogenetic richness is concentrated in relatively northern geographic areas (top) where precipitation is lower and temperatures are warmer (bottom). **(D)** Environmental suitability is concentrated in central and south-central Chile but extends into some parts of western Argentina (top). High suitability values trail higher temperatures with moderate precipitation (bottom). The base map for Argentina and Chile was downloaded from DIVA-GIS (https://diva-gis.org/data.html) with data provided by GADM under license CCBY (https://gadm.org/license.html).

**Fig 3:**
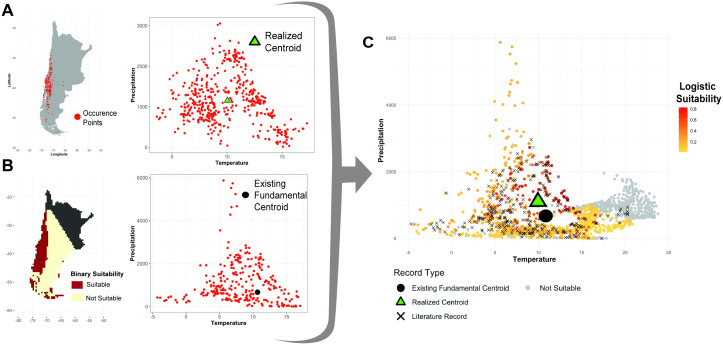
Overview of existing fundamental vs. realized niche in geographic and environmental space. **(A)** Occurrence points of *Oligoryzomys longicaudatus* from GBIF and our literature review in geographic and environmental space. The realized niche centroid (green triangle) is shown in environmental space. **(B)** Occurrence points are used in a maxent model to assess environmental suitability. The binary suitability (10 percent threshold) is shown in geographic and environmental space. The existing fundamental niche centroid (black circle) is shown in environmental space. **(C)** The existing fundamental and realized niches are shown together with the maxent logistic suitability predictions (yellow and red), points from the literature review (black **X)**, and the environmental areas in Chile and Argentina that were unsuitable (gray circle). The realized centroid was slightly warmer and wetter than the existing fundamental centroid. Some literature records fall outside the suitable area, which may be records of other *Oligoryzomys* species erroneous reported as *O. longicaudatus*. Areas with very high annual precipitation (in southernmost Chile) are reported as suitable, but literature records are lacking from these areas. The base map for Argentina and Chile was downloaded from DIVA-GIS (https://diva-gis.org/data.html) with data provided by GADM under license CCBY (https://gadm.org/license.html).

From GBIF we obtained 6167 occurrences attributed to *O. longicaudatus* (https://doi.org/10.15468/dl.ax8qe5). After cleaning, we retained 4523 with coordinates that were not flagged by Coordinate Cleaner. Using these occurrences, we built an approximate distribution for *O. longicaudatus* with the centroid at point -41.01°S, -72.42°W in San Manuel (Puerto Octay, Chile), which we used to calculate the distance to geographic center. The extremities of the distribution with recorded parasite or genetic data were recorded at -39.75°S, -62.25°W (western), -43.25°S, -74.25°W (eastern), -17.750°S, -69.25°W (northern), and -55.25°S, -68.75°W (southern) (Fig 2A and 2B). The environmental space occupied by *O. longicaudatus* with recorded parasite or genetic data reached a highest value of average annual temperature of 20.28°C and a lowest of -4.67 °C. The annual precipitation ranged from 0 (in the Atacama Desert) to 2961 mm (in Aysén, Chile). The most suitable area for *O. longicaudatus* was primarily recorded in Chile between latitudes -35° and -43° but spanned most of Chile and parts of southwestern Argentina (Fig 2D). Our suitability model which only included occurrences within the accepted distribution of *O. longicaudatus,* had a fundamental niche centroid at temperature = 10.61 °C and precipitation = 703.34 mm and a realized niche at temperature = 9.66 °C and precipitation = 1142.37 mm (Figs 3 and S1). The model based on all database and literature occurrences in Argentina and Chile had a fundamental niche centroid at temperature = 12.55 °C and precipitation = 670.22 mm, and a realized niche temperature = 10.621 °C and precipitation = 1143.31 mm (Figs 3 and S1).

### Genetic diversity

**Fig 4:**
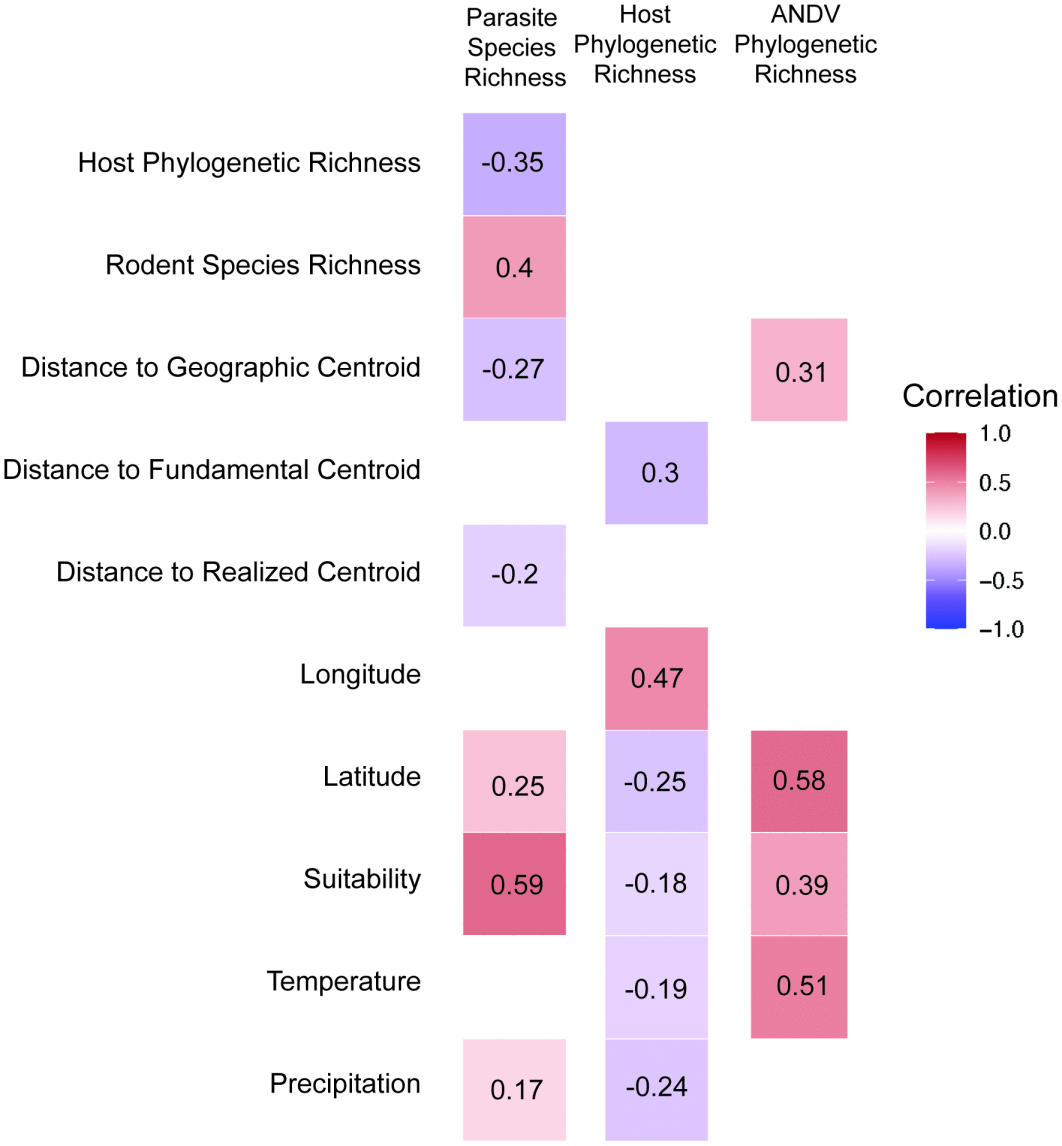
Significant spearman correlations between Parasite Species Richness, *Oligoryzomys longicaudatus* phylogenetic richness, and Andes Virus (ANDV) Phylogenetic Richness with environmental and geographic predictors. Relationships according to independent Spearman’s correlation are shown between measures of parasite species richness, ANDV phylogenetic richness, and *O. longicaudatus* phylogenetic richness and with environmental suitability, mean annual temperature, annual precipitation, distance to the geographic distribution centroid, rodent species richness, distance to the existing fundamental niche centroid, distance to the realized niche centroid, and latitude and longitude. The color bar indicates the direction and strength of the relationship with red indicating a positive relationship and blue a negative relationship where the gradient of the color corresponds to the significance of the relationship. Parasite species richness is significantly negatively correlated with distance to geographic centroid, distance to centroid of the realized niche, and significantly positively correlated with environmental suitability, rodent species richness, *O. longicaudatus* phylogenetic richness and annual precipitation. Environmental suitability shows a significant relationship with all variables. ANDV phylogenetic richness is most strongly correlated with temperature and latitude, with higher richness in higher latitudes where it is warmer. Phylogenetic richness of *O. longicaudatus* is most strongly correlated with longitude, exemplifying that outlier populations in Southern Chile and Argentina are more evolutionarily distinct than those in the central distribution.

We constructed a phylogenetic tree with all 237 cytochrome b sequences from *O. longicaudatus.* Nucleotide and haplotype diversity were calculated with 17 clusters of 14 individuals each. The highest phylogenetic richness for *O. longicaudatus* was recorded in the south-central portion of its distribution which extends into Argentina at 0.032, and the lowest value in the southernmost extremes of Chile 0.00002 (Fig 2B). The nucleotide diversity of *O. longicaudatus* ranged from 0.001 to 0.026 and was highest in middle latitudes (-40°) for the distribution of this species. The haplotype diversity of *O. longicaudatus* ranged from 0.495 to 0.978 and decreased with increasing latitude. Neither *O. longicaudatus* haplotype nor *O. longicaudatus* nucleotide diversity had a significant correlation with parasite and ANDV diversity (Fig 4; S1 Table). Phylogenetic richness of *O. longicaudatus* was negatively correlated with distance to the center of the existing fundamental niche with phylogenetic richness being high in the centroid and decreasing toward the edges of the niche (Fig 4; S1 Table; p = 6.016 × 10^-6^, ρ = -0.307). Phylogenetic richness of *O. longicaudatus* was also negatively correlated with environmental suitability (p < 0.01, ρ = -0.178), mean annual temperature (p = 0.005, ρ = -0.195), and annual precipitation (p = 0.0004, ρ = -0.242).

### Independent predictors of parasite and ANDV phylogenetic richness

Parasite species richness of *O. longicaudatus* varied markedly across its geographic range, with 35 parasite species recorded and two diversity hotspots near –40° and –35° latitude (Fig 2A). In independent correlation models, parasite species richness was most strongly positively correlated with environmental suitability followed by rodent species richness, suggesting that highly suitable areas with diverse host assemblages supported a more diverse parasite community (Fig 4, S1 Table). Parasite species richness was negatively correlated with distance to the geographic centroid and distance to the realized niche centroid of the host as well as *O. longicaudatus* phylogenetic richness (Fig 4; S1 Table). A weak positive association was also detected with annual precipitation, whereas no significant correlation was found with mean annual temperature, however areas of maximum parasite diversity occurred where temperatures exceeded 10 °C.

Similarly, phylogenetic richness of Andes virus (ANDV) exhibited a geographically structured pattern, ranging from 0.0001 in south-central Chile to 0.194 in Cauquenes (–35.75° S, –72.25° W; Fig 2C). In independent correlation models, ANDV phylogenetic richness increased with distance from the geographic center of *O. longicaudatus* distribution and with environmental suitability (Fig 4; S1 Table). In contrast, ANDV phylogenetic richness was unrelated to host phylogenetic richness. Temperature showed a positive correlation with ANDV phylogenetic richness, while precipitation and other variables showed no significant relationships (Fig 4; S1 Table).

### Multivariate models

Initial OLS regression models for both parasite species richness and ANDV phylogenetic richness exhibited significant spatial autocorrelation in residuals (parasite SR: Moran’s I = 0.42, p = 0.001; ANDV: Moran’s I = 0.22, p = 0.001), violating the assumption of independent errors and necessitating spatially explicit modeling approaches. The GAM for parasite species richness (negative binomial family, k = 50) explained 73.5% of the deviance with an adjusted R² of 0.68 (n = 153). The two-dimensional spatial smooth term was significant (edf = 24.8, F = 12.5, p < 0.001), suggesting substantial spatial structure in parasite richness patterns that was not captured by the measured environmental predictors alone. After accounting for this spatial structure, host phylogenetic richness showed a marginally significant negative association with parasite richness (β = -61.45, SE = 33.12, z = -1.86, p = 0.062). In contrast, the predictors that were significant in initial OLS models, including rodent species richness, distance to geographic centroid, and environmental suitability, lost statistical significance once spatial autocorrelation was included (all p > 0.12). Post-hoc Moran’s I testing confirmed that the spatial smooth term fully resolved residual spatial autocorrelation (Moran’s I = 0.05, p = 0.15). The GAM for ANDV phylogenetic richness (Gaussian family, k = 30) explained 73.5% of the deviance with an adjusted R² of 0.65 (n = 103). The spatial smooth term was highly significant (edf = 19.5, F = 8.9, p < 0.001), fully resolving residual spatial autocorrelation (post-hoc Moran’s I = -0.12, p = 0.96). After accounting for spatial structure, none of the environmental or host-related predictors significantly explained ANDV phylogenetic richness (all p > 0.19).

## Discussion

We analyzed host-parasite relationships through a novel framework that integrates parasite community data, host and viral genetic diversity, and environmental gradients across the geographic range of the medically important rodent *Oligoryzomys longicaudatus*. Our analyses revealed two contrasting patterns: (i) parasite species richness is concentrated in environmentally suitable, central regions with high rodent community diversity, whereas (ii) Andes virus (ANDV) phylogenetic diversity is highest toward geographic and environmental margins. After accounting for spatial autocorrelation, we found that geographic location itself, rather than specific environmental or host community predictors, was the dominant predictor of both diversity patterns. This does not indicate that environmental factors are unimportant, but rather that vicariance effects are so strong that simple bioclimatic averages cannot capture them independent of geography [81,85]. This suggests that geographic processes such as dispersal limitation, historical biogeography, and genetic drift, or fine-scale environmental heterogeneity may be more important than the environmental and host factors studied here.

### Geographic structure

A central finding from our spatially explicit analyses is that geographic location was the dominant predictor of both parasite species richness and ANDV phylogenetic diversity. Environmental and host-related predictors that appeared significant in bivariate correlations or standard OLS regression lost significance once spatial autocorrelation was properly modeled. This result should be interpreted in the context of the unique biogeography of our study system. The distributional range of *O. longicaudatus* extends along a narrow, latitudinally elongated corridor spanning over 2500 km from northern to southern Chile but is constrained to <200 km in width between the Pacific Ocean and the Andes Mountains [31,32]. In such geographically linear systems, spatial autocorrelation reflects real biological processes, often representing geographically structured environmental gradients, rather than being merely a statistical artifact [85].

An important interpretive question is whether the spatial smooth term in our model represents unmeasured factors entirely distinct from our predictors, or whether it captures fine-scale variation that our bioclimatic and host predictors inadequately represent. We argue the latter is more likely. The environmental predictors we used, mean annual temperature and precipitation, are spatially coarse summaries that cannot capture fine-scale habitat heterogeneity, seasonal climate dynamics, or critical ecological features such as *Chusquea bamboo* distribution which is a key resource for *O. longicaudatus* population dynamics [33,85].

The strong correlation between our environmental predictors and geographic location (e.g., precipitation increases southward with latitude) indicates that these factors are inherently spatial. When we include both precipitation (as a single average value) and the spatial smooth term in the same model, the spatial smooth dominates because it represents a more flexible surface that captures the coarse environmental variables, which we explicitly modeled plus additional spatially structured variation. This does not mean the environment is irrelevant to parasite species richness or viral diversity. The bivariate correlations illustrate clear environmental and host associations, that may operate across the spatial domain, resulting in strong spatial autocorrelation. As such, we argue that environmental effects are spatially structured and cannot be captured independent of geography [86]. The spatial structuring of environmental predictors is particularly evident in narrow, latitudinally elongated systems where environmental gradients are aligned along a single spatial axis [80,81]. Host-associated predictors, such as rodent community richness, are strongly spatially structured in Chile due to its unique biogeographic history [56]. For example, rodent species richness that overlaps with the distribution of *O. longicaudatus* is concentrated in the central-south portion of Chile, corresponding to a unique ecoregion with variables like higher productivity and environment that support higher mammal species richness [87].

For ANDV specifically, the spatial smooth may capture environmental effects on viral transmission dynamics that are not well-represented by annual averages. For example, seasonal temperature fluctuations that affect rodent density peaks, or drought patterns that drive host population crashes and subsequent changes in viral prevalence [35] are not captured by the average climatic values used here. For parasite richness, the spatial smooth likely captures host density patterns (which we did not directly measure but which correlate with suitability and precipitation), bamboo flowering events that drive population outbreaks, and microhabitat structure that affects ectoparasite survival [33,34].

### Parasite species richness

Parasite species richness was strongly geographically patterned, reaching peaks towards the center of the host’s range, and not significantly related to any host or environmental variable when accounting for spatial autocorrelation. This suggests that the apparent associations between rodent community richness and distance to geographic centroid in OLS models were at least partially confounded by unmeasured spatially structured factors. Unmeasured spatially structured factors associated with parasite species richness may be related to factors that proved significant in independent correlations, but without enough detail to show significance considering spatial structure. In independent correlations we found that parasite species richness was significantly higher in areas of high host environmental suitability, near the center of the host’s range, in areas with high rodent species richness, and in association with host phylogenetic richness. Elevated parasite species richness in these regions may reflect higher host densities, which peak in temperate southern forests of the Mediterranean and fluctuate with ENSO events [33,34]. This would support previous findings that higher host population densities increase opportunities for parasite transmission [20]. The association between parasite species richness and host phylogenetic richness parallels findings in humans, where parasite species richness correlates with cultural diversity metrics such as language and religion [88].

In wildlife, environmental suitability could also track genetic diversity [89], underscoring links among phylogenetic richness, parasite species richness, and suitability, and suggesting possible interdependence. We also found that parasite species richness in *O. longicaudatus* was positively correlated with rodent species richness. This reflects findings that *O. longicaudatus* shares ectoparasites with many other rodent species in Chile [30] and exemplifies how a diverse host community can increase parasite diversity within a single host through local parasite sharing. Furthermore, *O. longicaudatus* underwent a postglacial expansion (26,000 to about 13,000 years ago) from its ancestral distribution in the southern temperate forests of Chile (40°S) [32,90]. This ancestral distribution aligns with present areas of high suitability, host phylogenetic richness, and parasite species richness. The evolutionary history of *O. longicaudatus* in southern Chile [32] may provide context for the present-day trends we found. The expansion of *O. longicaudatus* out of southern Temperate forests could have facilitated the present-day pattern in which there is higher parasite species and phylogenetic richness in the ancestral distribution, due to having had more time to develop host-parasite relationships, than in areas where the species has more recently expanded [16].

A limitation for our estimation of parasite species richness is that areas with high parasite species richness could be related to relative sampling effort, especially given that higher host density, and therefore the ease to sample hosts, has been reported in the areas where we found high parasite species richness We conducted species accumulation analysis, which suggested adequate overall sampling, but spatial bias remains possible. The spatial smooth term in GAMs accounts for spatially structured sampling heterogeneity and if sampling effort is geographically patterned, the smooth captures that pattern regardless of whether it reflects biology or methodology. We cannot definitively disentangle biological patterns from potential sampling artifacts without standardized, spatially balanced parasite surveys with equal effort across the host range and reporting of negative results. Nevertheless, the concentration of parasite richness in the ancestral range of the host is consistent with the ecological and evolutionary history of *O. longicaudatus* and similar patterns have been documented in other systems where sampling bias is less of a concern [91]. Moreover, the marginal significance of host phylogenetic diversity even after spatial correction, provides some evidence for biological drivers beyond geographic location alone. From a surveillance perspective, central regions of the *O. longicaudatus* distribution with high parasite richness, regardless of whether this reflects purely biological processes or includes sampling artifacts, represent areas where diverse multi-host parasite communities circulate. These core zones of parasite species richness, which are at both the center of the rodent’s distribution and niche, may act as sources for cross-species spillover and warrant targeted surveillance for zoonotic pathogens beyond ANDV, particularly parasites shared among rodent species [30,91,92]

### ANDV diversity

In contrast to parasite richness, ANDV phylogenetic richness was highest at the edges of the host’s distribution, particularly in the northern half of the hosts distribution (35°–30°S), and showed positive bivariate correlations with temperature, distance from geographic range center, and environmental suitability. Spatially explicit GAM analysis revealed that none of these environmental predictors remained significant after accounting for spatial autocorrelation, only the spatial smooth term was significant. This pattern is more consistent with neutral geographic processes than with environmental selection. Specifically, peripheral populations may experience genetic drift in isolated populations where stochastic processes accelerate divergence, limited viral gene flow due to the narrow geographic corridor and dispersal barriers, and isolation-by-distance effects where distance reduces connectivity regardless of environmental similarity [93,94]. The narrow distribution of *O. longicaudatus*, constrained between the Pacific Ocean and the Andes Mountains, limits dispersal opportunities and likely contributes to strong phylogeographic structure in both host and viral populations. Nevertheless, environmental variables that we did not capture in our model may still influence ANDV diversity. Given that *O. longicaudatus* is known to be sensitive to dry, warm conditions, these patterns suggest that ANDV diversity has accumulated in areas that the species can tolerate, but may not be optimal [95,96]. This could reflect populations under suboptimal climatic conditions [97] where they may have lower reproductive success and be more susceptible to infections, as demonstrated in other disease systems such as white-nose syndrome in bats [98].

We also emphasize important caveats regarding viral diversity inference. Our analysis is based on approximately 30 sequences from a single genomic segment (S segment), which constrains resolution for detecting adaptive evolution or fine-scale diversification patterns. Diversity in ANDV isolated from *O. longicaudatus* may also be influenced by the circulation of other viral subspecies of *Orthohantavirus andense* in various hosts in countries bordering or near northern Chile [99,100]. There are 13 subspecies and 29 hosts of *Orthohantavirus andenese* that have been molecularly identified circulating in South America, many of which were first detected in Brazil, Argentina, Paraguay, and Uruguay [99]. Nevertheless, the known secondary host species associated with ANDV in Chile (*Abrothrix olivacea, Abrothrix longipilis*, *Phyllotis darwini*, and *Loxodontomys micropus*) overlap with *O. longicaudatus* in geographic range in the south-central portions of Chile where there is low ANDV phylogenetic richness from the principal host [30,101]. This suggests that cross-species spillover of ANDV occurs in areas where viral phylogenetic richness in the primary host is low, contradicting the expectation that viral diversity would be higher when there are more host species [102].

The lack of correlation between ANDV diversity and host genetic diversity could indicate an evolutionary distinction between host and viral trajectories. This is not entirely unexpected in hantaviruses, which experience rapid mutation rates and localized adaptation, driven more by stochastic processes and ecological isolation than by host ecology [103,104], but contrasts with findings in the rabies virus system where host genetic diversity tracked viral diversity [105]. These findings align with a broader pattern observed in disease systems, in which macroparasite (e.g., helminth, ectoparasite) communities track host ecology, while microparasite (e.g., viruses) diversity is more sensitive to environmental filters and geographic structure [106]. Additionally, it is important to consider the geographic patterns of ANDV diversity in the context of the temporal scale. In contrast to parasite faunas, which reflect deep evolutionary and ecological histories, intraspecific viral diversity can turn over rapidly within decades or even years. Under this interpretation, temporal asymmetry between parasite and viral diversification could be a potential organizing mechanism that predicts the geographic mismatch we observe. Together, these patterns suggest that spatial processes, particularly isolation-by-distance and ecological compartmentalization, may be the primary mechanisms through which rapid viral diversification leaves a geographic signature that is fundamentally distinct from the slower-accumulating parasite community richness.

### Implications in hantavirus ecology

Our findings reveal a geographic mismatch between viral diversity and human disease risk with implications for surveillance strategies. The Mediterranean ecoregion of Chile, at the northern extent of the host’s range, was found as a nucleus of ANDV diversification with the highest phylogenetic richness in the Maule region. In Chile, rodent ANDV seroprevalence was also highest in the Maule region while human cases are highest in more southern regions of Aysén and Los Lagos [35]. This may indicate that high viral diversity and rodent seroprevalence are not necessarily facilitators for spillover in this system. Human spillover cases in Maule, where ANDV phylogenetic diversity and rodent seroprevalence are high, may also be lower than incidences in the south due to infrequent encounters between rodents and humans rather than the absence of risk [107,108]. Additionally, human spillover cases may be low in regions with high viral seroprevalence and diversity because mutations leading to higher diversity are associated with low transmissivity and virulence, limiting disease emergence in these environments [109]. Future studies integrating spatially-explicit sampling of rodent biodiversity and abundance, human population density, viral prevalence (not just genetic diversity), and fine-scale ANDV genomic variation across multiple segments [100] are an important future research line to determine why human infection with hantavirus in Chile is concentrated in southern regions, where host seroprevalences and viral diversity are comparatively lower [35].

### Host distribution and environmental suitability

The complete distribution of *O. longicaudatus* extends from latitude 27° to 54° S in Chile and into Patagonian and Temperate forests of Argentina [32,71,90,110]. The distribution of *O. longicaudatus* is dynamic and exhibits strong seasonal and climatic variability, disappearing from mesic areas of its distribution during drier years [95], increasing during ENSO events [33,34], and has greater water requirements than its sympatric species [96]. Since 2010, Chile has been experiencing a megadrought [111], which has undoubtedly impacted *O. longicaudatus* populations given its reliance on humid environments. This dynamic nature of *O. longicaudatus* in response to climate variation is not captured in our suitability model but will have implications on this species’ persistence in the more northern extents of its range. Despite the limitations of suitability models, our results show that predicted environmental suitability largely matches this established distribution from the literature. This consistency holds even when incorporating published literature records outside the accepted range, underscoring agreement between predicted suitability and published distributions. The records we obtained in our literature review which were attributed to *O. longicaudatus* in the most northern regions of Chile and northwestern of Argentina likely belong to *O. andinus* or *O. flavescens* [110,112,113]. Using both molecular and morphological identification for *O. longicaudatus* in reporting scientific findings may be necessary, since reported discrepancies in its distribution and potential impacts of recent extreme climate shifts on this species will have implications for public health risk of Andes hantavirus [110]. These taxonomic uncertainties in peripheral populations may also contribute to apparent elevated ANDV diversity if viral sequences attributed to *O. longicaudatus* come from cryptic congeners, reinforcing the need for integrated host-virus sampling with confirmed host species identification.

### Implications for disease ecology and surveillance

The contrasting geographic and environmental correlates of parasite and viral diversity in *O. longicaudatus,* where there is high parasite diversity in the center versus high viral diversity in the margins have implications for disease ecology and zoonotic risk. For instance, core areas with high environmental suitability for the host and high parasite richness facilitate the circulation of diverse parasites and may act as sources for spillover to other species [114,115]. Central areas of high suitability, where local host population density is higher [33,34], may also serve as local hotspots of ANDV transmission due to increased interactions between *O. longicaudatus* individuals as evidenced by microgeographic genetic structure [116]. Meanwhile, peripheral regions with high viral diversity may be hotspots for the emergence of novel viral strains [117–119]. This incongruity suggests that surveillance strategies should be geographically stratified in two areas: (i) central high-suitability regions warrant monitoring for spillover risk and transmission intensity (public health focus), while (ii) peripheral regions with high viral diversity should be targeted for genomic surveillance to detect emergence of novel hantavirus variants with uncertain epidemic and pandemic potential.

## Conclusion

By contrasting interspecific parasite richness and intraspecific viral diversity within a single host species, this study contributes to existing eco-evolutionary theory [37] by illustrating that parasite community vs. viral diversity responds to distinct ecological and geographic constraints. Parasite richness was concentrated in central, environmentally suitable regions while viral diversity peaked in geographic and environmental margins. Notably, geographic location itself emerged as the dominant predictor of both patterns, suggesting that environmental effects in this system are so strongly geographically structured that they cannot be captured independently of geographic processes. This geographic dominance may reflect complex interactions among dispersal limitation, historical biogeography, and geographically continuous environmental variation that extend beyond bioclimatic averages.

The geographic mismatch between parasite richness and viral diversity hotspots has implications for disease surveillance and risk assessment strategies. Our findings suggest that regions of highest environmental suitability, where transmission potential peaks, do not coincide with areas of greatest hantavirus genetic diversity. This distinction underscores the need for geographically targeted surveillance approaches that differentiate between central transmission hotspots which require intensive public health monitoring and peripheral evolutionary hotspots which demand genomic surveillance for emerging viral lineages. Adopting this geographically explicit, eco-evolutionary framework that bridges macroecological patterns of parasite community assembly with microevolutionary dynamics of viral diversification provides essential insights for anticipating disease emergence and designing targeted surveillance strategies across heterogeneous host geographic ranges.

## Supporting information

S1 Table Article information associated with *Oligoryzomys longicaudatus* records from our literature review. For each study, the Author Year, title, and a Study ID are reported. When available the study ID is the DOI or PMID. The ‘Author Year’ field can be used as an identifier to find full study information from reports of ANDV, parasites, and genetic data.
(XLSX)

S2 Table Results of parasites found associated with *Oligoryzomys longicaudatus* from our literature review. It reports the Author, Year of the paper, the start and end year of the study, the type of parasite, the parasite name, the method of analysis, the country, region, locality, and the latitude and longitude.
(XLSX)

S3 Table Metadata associated with S segment sequences of Andes virus available through GenBank. Data reported include locality, region, country, reference, latitude, longitude, collection period, and GenBank Accession number.
(XLSX)

S4 Table The data associated with cytochrome b sequences from *Oligoryzomys longicaudatus* available through GenBank collected from our literature review. Data reported include reference, locus, GenBank accession, country, region, locality, longitude, and latitude.
(XLSX)

S5 Table Results from Spearman’s rank correlation. Relationships between *O. longicaudatus* haplotype diversity*, O. longicaudatus* nucleotide diversity, *O. longicaudatus* phylogenetic richness, environmental suitability, average annual temperature, annual precipitation, distance to center of range, distance to fundamental niche centroid, and distance to realized niche centroid with ANDV phylogenetic richness, parasite species richness, and *O. longicaudatus* phylogenetic richness. Significant relationships are in bold.
(PDF)

S1 Fig Environmental Suitability of *Oligoryzomys longicaudatus* according to all vs. accepted distribution records. The environmental suitability *for O. longicaudatus* was assessed using maxent in two separate analyses. In one (shown on the left) we included all records from the literature and GBIF attributed *to O. longicaudatus* that were within Chile and Argentina. In the other (on the right) we used records matching the distribution from IUCN and published literature for the species. The suitability is shown first as continuous values which can be from 0 to 1, then as a binary using a 10 percent training threshold, and lastly is projected into environmental space (i.e., annual precipitation and average annual temperature) with the continuous value. Using all records from Chile and Argentina expands the suitable area of the species in north, south, and east directions. The base map for Argentina and Chile was downloaded from DIVA-GIS (https://diva-gis.org/data.html) with data provided by GADM under license CCBY (https://gadm.org/license.html).
(PDF)
