# Supplementary material for: Contrasting geographic patterns of parasite and hantavirus diversity in the rodent Oligoryzomys longicaudatus (Rodentia, Cricetidae)
Source: PLoS Negl Trop Dis. 2026 Jun 26;20(6):e0014424. doi: 10.1371/journal.pntd.0014424 (PMC13345464; doi:10.1371/journal.pntd.0014424)
Supplement: S5 Table — Relationships between O. longicaudatus haplotype diversity, O. longicaudatus nucleotide diversity, O. longicaudatus phylogenetic richness, environmental suitability, average annual temperature, annual precipitation, distance to center of range, distance to fundamental niche centroid, and distance to realized niche centroid with ANDV phylogenetic richness, parasite species richness, and O. longicaudatus phylogenetic richness. Significant relationships are in bold. (PDF) [file pntd.0014424.s005.pdf]

	ANDV Phylogenetic richness	Parasite Species Richness	*O. longicaudatus* Phylogenetic Richness
Haplotype diversity	p=0.729, ρ=-0.118	p=0.77, ρ=-0.086	p= 0.959, ρ=-0.13
Nucelotide diversity	p=0.356, ρ=-0.309	p=0.362, ρ= -0.264	p= 0.479, ρ=0.184
O. longicaudatus Phylogenetic Richness	p=0.803, ρ=-0.25	**p= 7.57e-06, ρ=-0.345**	
Parasite Species Richness	p=0.24, ρ=-0.111		p= 7.57e-06, ρ=-0.345
Environmental suitability	**p=1.643e-05, ρ=0.392**	**p<2×10^-16, ρ=0.588**	**p<0.01, ρ= -0.178**
Average annual Temperature	**p= 4.981e-09, ρ=0.514**	p= 0.0204, ρ=0.149	**p=0.005, ρ= = -0.195**
Annual Precipitation	p= 0.133, ρ=-0.142	**p= 0.008, ρ=0.169**	**p=0.0004, ρ=-0.242**
Distance to center of range	**p=0.0007, ρ=0.3128**	**p=2.95e-05, ρ=-0.265**	p=0.269, ρ=0.077
Distance to centroid of fundamental Niche	p=0.177, ρ = -0.127	p= 0.741, ρ=-0.021	**p=6.016e-06, ρ=-0.307**
Distance to centroid of realized niche	p=0.2035, ρ=-0.119	**p=0.002, ρ=-0.202**	p= 0.524, ρ = -0.044
Rodent Species Richness	p=0.459, ρ=-0.071	**p=2.49e-09, ρ=0.401**	p= 0.039, ρ = -0.151

Table S5. Results from Spearman's rank correlation for relationships between *O. longicaudatus* haplotype diversity, *O. longicaudatus* nucleotide diversity, *O. longicaudatus* phylogenetic richness, environmental suitability, average annual temperature, annual precipitation, distance to center of range, distance to fundamental niche centroid, and distance to realized niche centroid with ANDV phylogenetic richness, parasite species richness, and *O. longicaudatus* phylogenetic richness. Significant relationships are in bold.
