# Supplementary material for: Contrasting geographic patterns of parasite and hantavirus diversity in the rodent Oligoryzomys longicaudatus (Rodentia, Cricetidae)
Source: PLoS Negl Trop Dis. 2026 Jun 26;20(6):e0014424. doi: 10.1371/journal.pntd.0014424 (PMC13345464; doi:10.1371/journal.pntd.0014424)
Supplement: S1 Fig — The environmental suitability for O. longicaudatus was assessed using maxent in two separate analyses. In one (shown on the left) we included all records from the literature and GBIF attributed to O. longicaudatus that were within Chile and Argentina. In the other (on the right) we used records matching the distribution from IUCN and published literature for the species. The suitability is shown first as continuous values which can be from 0 to 1, then as a binary using a 10 percent training threshold, and lastly is projected into environmental space (i.e., annual precipitation and average annual temperature) with the continuous value. Using all records from Chile and Argentina expands the suitable area of the species in north, south, and east directions. The base map for Argentina and Chile was downloaded from DIVA-GIS (https://diva-gis.org/data.html) with data provided by GADM under license CCBY (https://gadm.org/license.html). (PDF) [file pntd.0014424.s006.pdf]

Supplementary

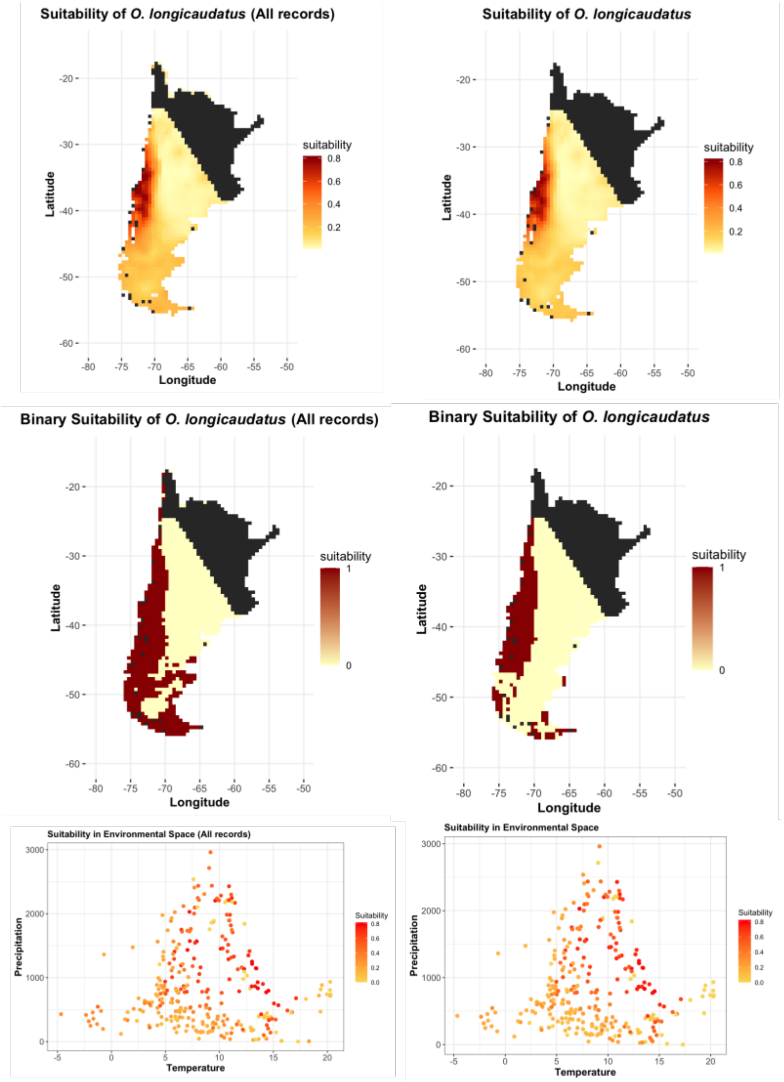

Figure S1. Environmental Suitability of *Oligoryzomys longicaudatus* according to all vs. accepted distribution records. The environmental suitability for O. longicaudatus was assessed using maxent in two separate analyses. In one (shown on the left) we included all records from the literature and GBIF attributed to *O. longicaudatus* that were within Chile and Argentina. In the other (on the right) we used records matching the distribution from IUCN and published literature for the species. The suitability is shown first as continuous values which can be from 0 to 1, then as a binary using a 10 percent training threshold, and lastly is projected into environmental space (ie. annual precipitation and average annual temperature) with the continuous value. Using all records from Chile and Argentina expands the suitable area of the species in north, south, and east directions.
